# Supplementary material for: Tetrapod limb and sarcopterygian fin regeneration share a core genetic programme
Source: Nat Commun. 2016 Nov 2;7:13364. doi: 10.1038/ncomms13364 (PMC5097137; doi:10.1038/ncomms13364)
Supplement: Supplementary Information — Supplementary Figures 1 - 5 and Supplementary Tables 1 - 3 [file ncomms13364-s1.pdf]

Supplementary Figures

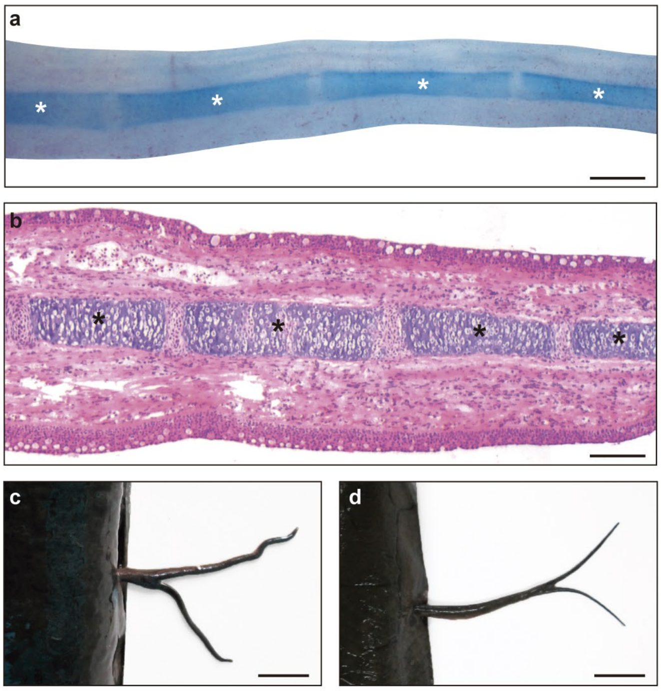

Supplementary Figure 1. Normal lungfish fin morphology and duplicated fins. a, Alcian blue staining of the South American lungfish fin endoskeleton showing a series of distinct cartilaginous units, or mesomeres (white asterisks), which extend distally and form a central axis. **b**, Haematoxylin and eosin stained sections showing mesomeres (black asterisks). **c**, **d**, Examples of duplicated fins from wild-caught specimens (n = 7, biological replicates). Scale bars of 0.5 mm (**a**, **b**) and 10 mm (**c**, **d**).

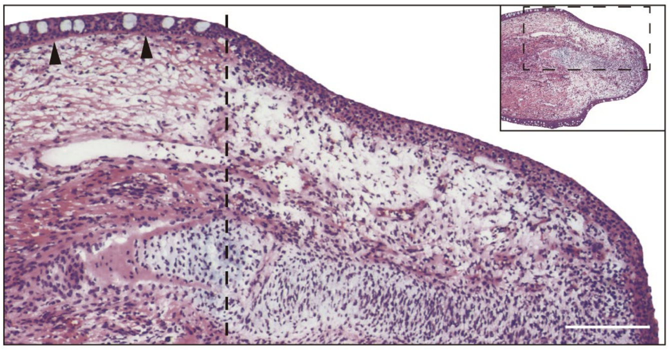

Supplementary Figure 2. Loss of basement membrane during blastema formation. Haematoxylin and eosin stained 3 wpa blastema. The basement membrane is denoted (black arrowheads) underlying the epithelium proximal to the amputation plane (dashed line). Inset shows the whole blastema. Scale bar of 0.25 mm.

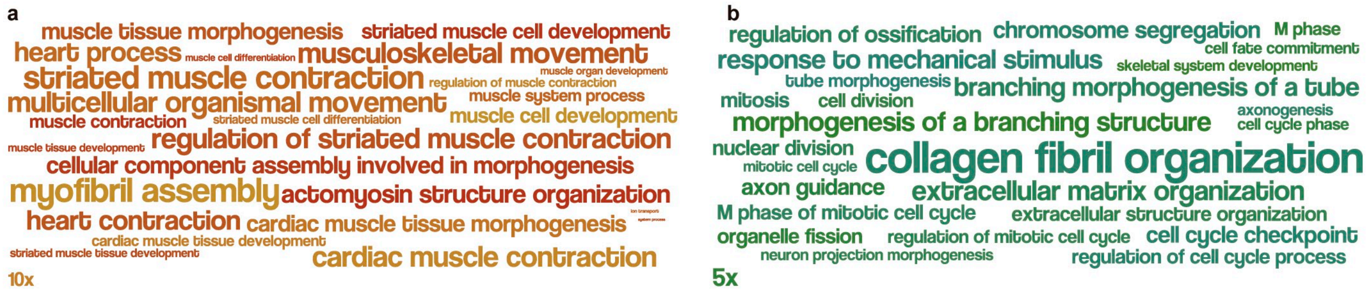

Supplementary Figure 3. Gene ontology of South American lungfish fin regeneration. Word clouds showing 25 top GO-enriched terms among downregulated (**a**) or upregulated (**b**) genes. Fold enrichment is denoted by the size of words, with a fold enrichment inset scale of 10x (**a**) and 5x (**b**).

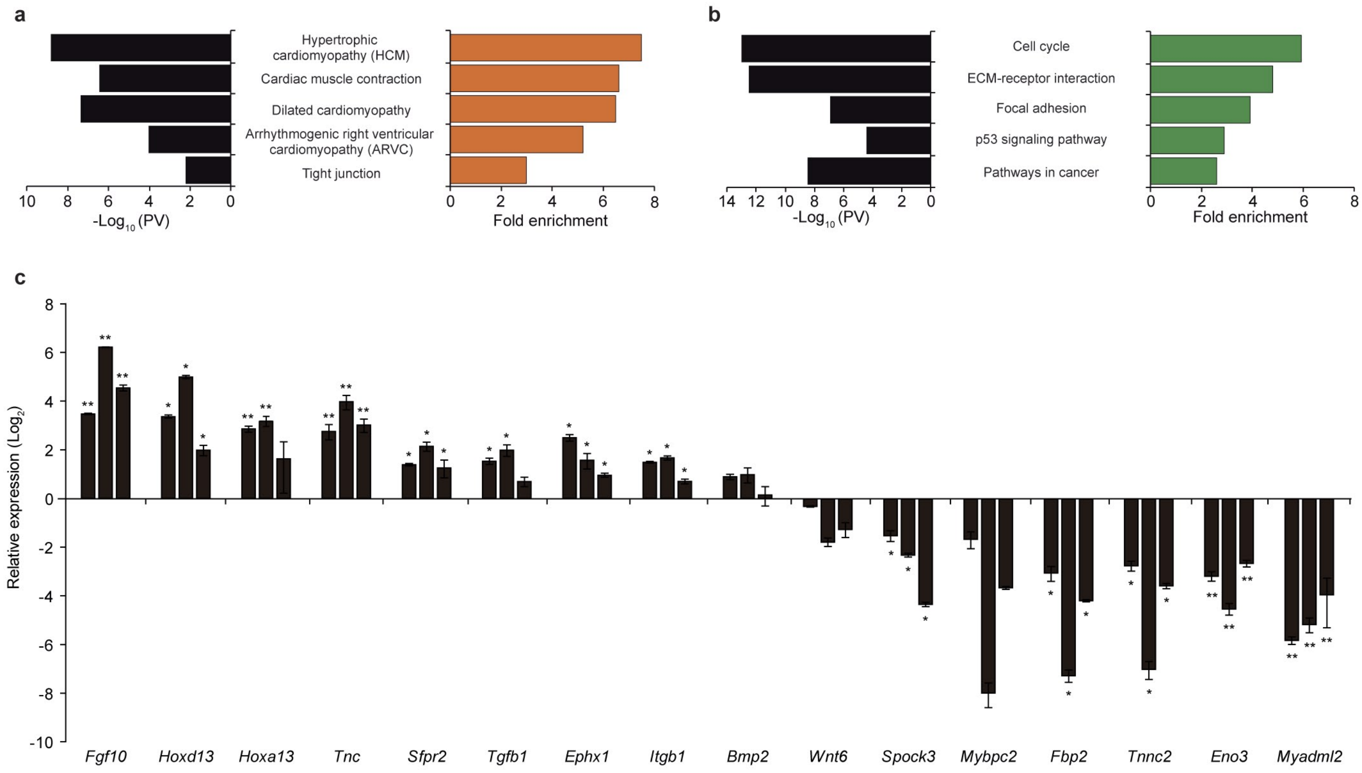

Supplementary Figure 4. qPCR analysis of selected up and downregulated genes. **a**, Four of the top 5 KEGG pathways among downregulated genes are related to muscle function. **b**, Top 5 KEGG pathways among upregulated genes. KEGG pathways are shown relative to fold enrichment (color bars) and -log10 PV (black bars). **c**, A total of 9 upregulated and 7 downregulated genes identified from digital differential gene expression analysis were confirmed by qPCR. Relative expression ($2^{-\Delta\Delta CT}$) is shown in a log2 scale and each gene was analyzed in biological and technical triplicates (n = 18: 6 biological, 3 technical replicates each). Data are mean ± s.d.; PV, p value; ** PV < 0.01 and *PV < 0.05 using Student's t-test.

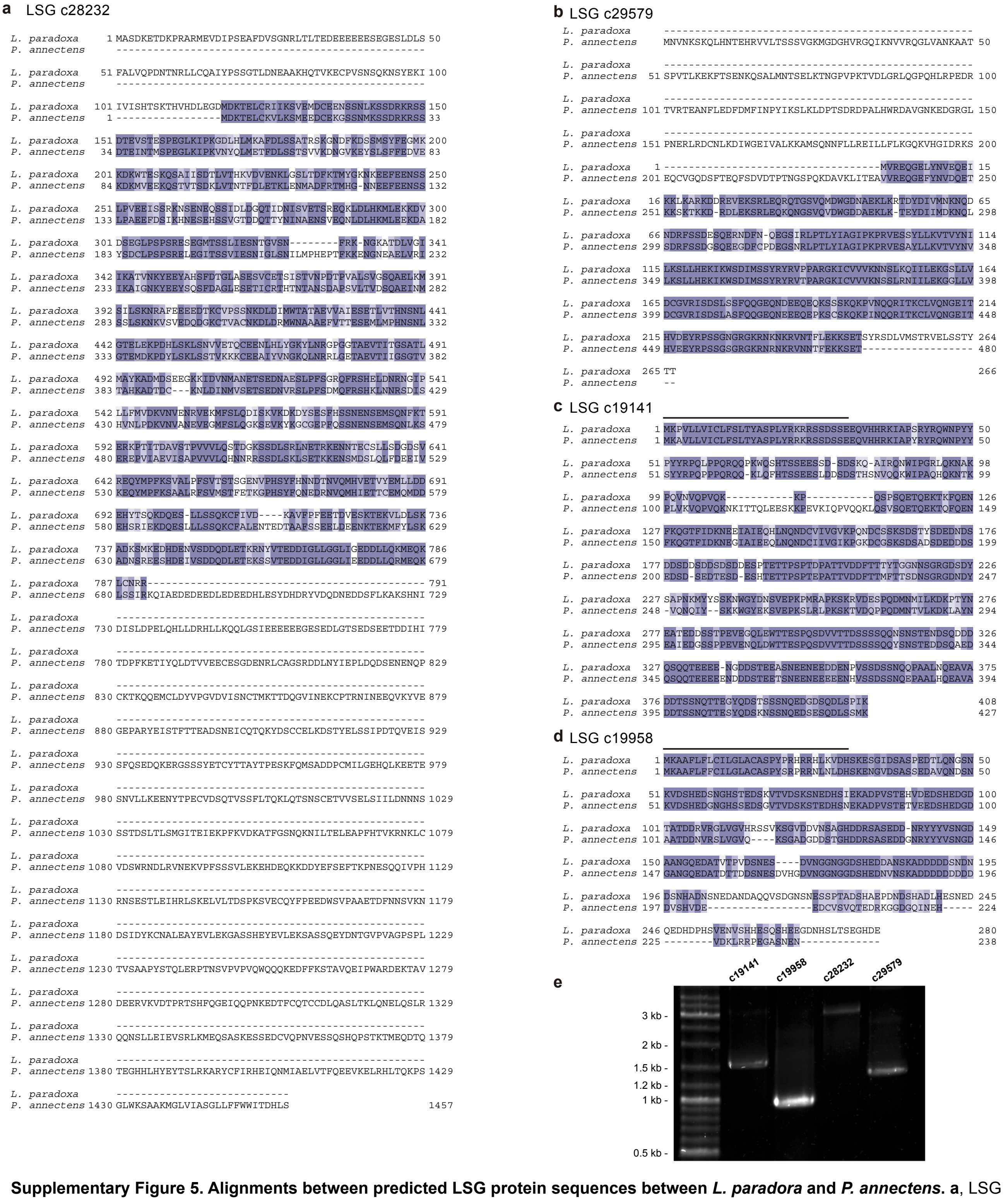

Supplementary Figure 5. Alignments between predicted LSG protein sequences between *L. paradora* and *P. annectens*. a, LSG c28232; **b**, LSG c29579; **c**, LSG c19141 and; **d**, LSG c19958. **e**, Agarose gel showing PCR-amplified LSGs from heart cDNA (c28232, 3038 bp; c19958, 933 bp; c19141, 1561 bp and; c29579, 1292 bp). Predicted signal peptide sequences marked with a black line on top (**c**, **d**).

Supplementary Tables

Supplementary Table 1. Statistics of the blastema reference transcriptome.

Total number of high quality assembled paired-end reads	113,896,894
Number of created contigs	122,014
Number of bases in contigs	108,403,587
Average length (bp)	889
N50	2,468
Longest contig (bp)	22,351
Number of contigs > 200 bp	122,014 (100%)
Number of contigs > 1 Kb	28,992 (23.8%)
Average ORF size (GenemarkS) (bp)	426
Number of predict proteins (GenemarkS)	29,699
Number of predict proteins full-length (GenemarkS)	14,246
Number of proteins with homology (e-value 10^{-10}/UniProt Metazoan subset)	37,397
Number of proteins with homology (e-value 10^{-10}/Refseq Human subset)	28,844
Number of proteins with GO annotations	33,254

Supplementary Table 2. Statistics of NRF RNA-seq runs and FB RNA-seq runs.

	NRF 1	NRF 2	NRF 3	FB 1	FB 2	FB 3
Total reads	27,812,392	16,821,701	25,361,601	22,341,526	22,235,658	29,292,327
Trimmed reads	27,810,568	16,820,687	25,360,097	22,339,829	22,234,313	29,290,540
Uniquely Mapped reads	22,152,046	13,370,262	20,408,666	17,763,155	17,747,591	23,252,812
Non-specific mapped reads	3,873,976	2,422,386	3,437,626	3,273,050	3,199,768	4,358,224
Uncounted reads	1,784,546	1,028,039	1,513,805	1,303,624	1,286,954	1,679,504
Counted reads	26,026,022	15,792,648	23,846,292	21,036,205	20,947,359	27,611,036

Supplementary Table 3. Pearson correlation coefficients among NRF and FB RNA-seq replicates.

	NRF 1	NRF 2	NRF 3	FB 1	FB 2	FB 3
Counted reads	26,026,022	15,792,648	23,846,292	21,036,205	20,947,359	27,611,036
NRF 1						
NRF 2	98.30%					
NRF 3	97.90%	97%				
FB 1	93.50%	96.20%	92.30%			
FB 2	92.50%	95.80%	91.50%	99.80%		
FB 3	93.50%	95.90%	92.00%	98.80%	98.30%	
